# Open globe injury with an interesting intra-ocular foreign body

**DOI:** 10.3205/oc000068

**Published:** 2017-07-07

**Authors:** Ekjyot Gill, Matthew Shulman, Sid Schechet, Lawson Grumbine

**Affiliations:** 1 University of Maryland, Baltimore, MD, USA

## Abstract

**Introduction:** Cases of penetrating ocular trauma due to osseous material are limited, so reported incidents are valuable in determining outcomes and proper treatment courses.

**Case description:** We report a case of an open globe injury of the left eye with an intraocular foreign body occurring after a firework exploded in the hand of a 22-year-old man. The patient presented with light perception vision in the injured eye with a full-thickness limbal laceration and dense hyphema obscuring fundoscopy. CT scan revealed a hyperdense foreign body juxtaposed to the lens. Immediate surgical intervention to repair the globe rupture revealed a defect in the anterior capsule and small, white objects in the posterior chamber that were promptly removed. Pathologic investigation determined these fragments to be cortical bone likely from the patient’s phalanges.

**Results and discussion:** There was no evidence of endophthalmitis or keratitis from time of injury to the five-month follow-up, suggesting that the risk of infection may be low and therefore it may be reasonable to manage these injuries with a period of observation.

## Introduction

Intraocular foreign bodies (IOFB) typically occur after high-velocity injuries and consist of either organic or inorganic materials. While both subsets of materials may cause orbital or intraocular complications from direct trauma, retained organic materials carry an increased risk of post-traumatic orbital infections [1], [2]. Early management of IOFBs is critical to reduce morbidity and should be tailored to the composition of the IOFB in question. This report describes a case of penetrating ocular trauma caused by an unusual foreign body: a fragment of the patient’s phalanges.

## Case description

**Figure 1:**
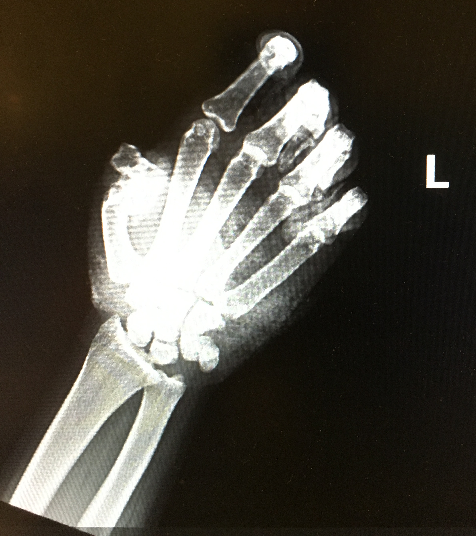
Imaging of hand after loss of distal fingers

Following the explosion of a firework grasped in his left hand, a 22-year-old male suffered loss of the distal portions of several fingers (Figure 1 (Fig. 1)) in addition to sustaining diffuse lacerations throughout his left upper extremity and face. Immediately after the event, the patient described a painful loss of vision in his left eye. 

**Figure 2:**
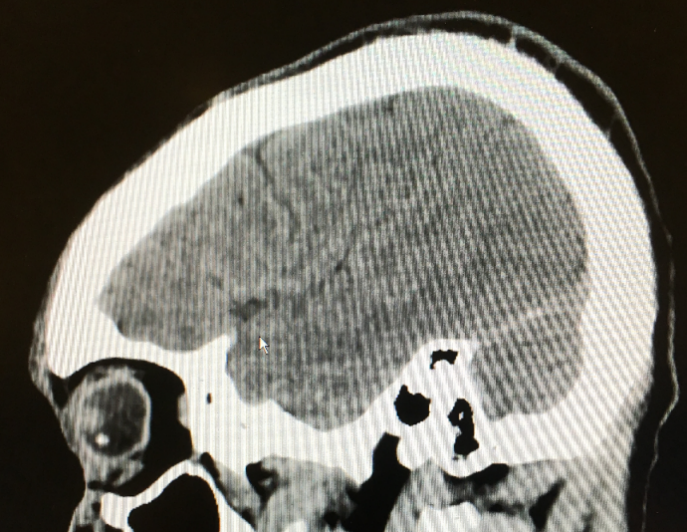
A foreign body seen within the globe on CT-imaging

On presentation to the Emergency Department, visual acuity in the left eye was light perception only. Slit lamp examination revealed laceration of the temporal cornea along the limbus with prolapsed uveal tissue. A dense “eight-ball” hyphema obscured any view of the posterior pole. CT of the face and orbits demonstrated a hyperdense foreign body adjacent to the lens (Figure 2 (Fig. 2)). The fellow eye was uninjured. After prompt administration of prophylactic intravenous antibiotics, the patient was taken to the operating room for repair of his ruptured left globe. 

**Figure 3:**
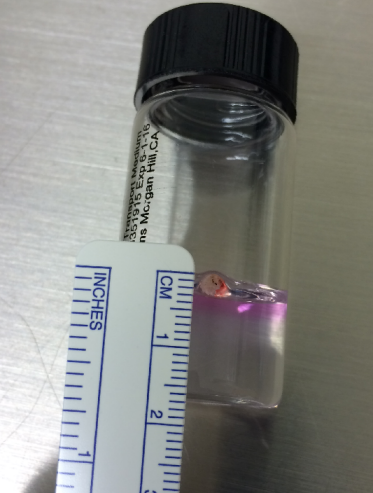
The removed foreign body

Surgical exploration of the left eye revealed a 5 mm full-thickness corneal-limbal laceration from the 2:30 to 4:00 o’clock position that was sutured closed. Two clock-hours of iridodialysis were noted after wash-out of the dense hyphema, and the anterior capsule was found to be violated with two dense white bodies noted in the anterior vitreous. A lensectomy and anterior vitrectomy were performed with removal of the two foreign bodies, which were then sent to pathology for evaluation. The largest IOFB was approximately 3.5 mm in greatest dimension (Figure 3 (Fig. 3)) and the second measured around 2 mm. As the composition of the foreign bodies were unknown at the time, intravitreal vancomycin and ceftazidime were administered.

Postoperatively, the patient developed hemorrhagic choroidal effusions, an open-funnel retinal detachment and proliferative vitreoretinopathy with continued light perception vision. Less than two months after initial repair, the patient underwent a left pars-plana vitrectomy with silicone oil placement. Five months later, the retina remained detached with vision improved to hand motion and without evidence of endophthalmitis.

## Discussion

The composition of the IOFB often dictates the visual outcome and risk of infection following trauma. Loss of vision with inorganic IOFBs is primarily a result of the initial trauma, whereas endophthalmitis is the leading cause of vision loss from organic IOFBs [1], [2]. Typically, inorganic materials like glass, stone, and plastic are better tolerated in the eye than organic material due to their inert nature. Regardless of IOFB composition, however, all patients with a penetrating ocular injury should receive prophylactic antibiotic therapy due to the high incidence of secondary infections [1].

While surgical removal is indicated for all retained organic material, inorganic materials may not warrant removal unless complications arise or if there is anterior migration [1]. Notably, there are three inorganic materials that must be removed: copper, due to its pro-inflammatory response; lead, due to the potential for systemic toxicity; and iron, which can cause siderosis [1], [3]. A retrospective study on patients who suffered globe ruptures during Operation Iraqi Freedom suggests that a delay in removal of inert substances does not increase complication rates of endophthalmitis [4].

Cases of bone fragment IOFB injury are exceedingly rare, and there is no significant data on post-injury infection risk in these cases. Only two cases of penetrating ocular injuries due to bone have been reported in the literature. The first case involved blunt facial trauma resulting in an orbital floor fracture with the patient’s own bone fragment penetrating the sclera, choroid, and retina. The bone fragment was left in place and monitored conservatively with no resultant signs of infection or complications three months later [2]. In the second case, an explosive detonated in a soldier’s hand resulting in penetrating ocular injury from projectile cortical bone fragments. A pars-plana vitrectomy was performed to remove the bone fragments, and the patient did not develop infection or significant complications at follow-up [5].

In our case, a lensectomy and anterior vitrectomy were performed with removal of two IOFBs. Their precise composition was not known at the time of surgery, so intravitreal antibiotics were administered prophylactically due to concern for organic material. While data on penetrating ocular trauma due to bone is limited, our findings, along with one of two other similarly reported cases, suggest that the risk of infection may be low. It seems reasonable to manage these injuries with a period of observation rather than proceeding to early vitrectomy for removal of the foreign body.

## Notes

### Acknowledgments

Thank you to the patient in this report for allowing us to share his case and thank you to the University of Maryland R Adams Cowley Shock Trauma Center and the University of Maryland Ophthalmology Department. 

### Competing interests

The authors declare that they have no competing interests.
